# Supplementary material for: Comparative Transcriptome Analysis of Arabidopsis Seedlings Under Heat Stress on Whole Plants, Shoots, and Roots Reveals New HS-Regulated Genes, Organ-Specific Responses, and Shoots-Roots Communication
Source: Int J Mol Sci. 2025 Mar 10;26(6):2478. doi: 10.3390/ijms26062478 (PMC11942352; doi:10.3390/ijms26062478)
Supplement: Supplementary file 1 [file ijms-26-02478-s001.zip › Additional file 1/Supplemental Fig. 1.pdf]

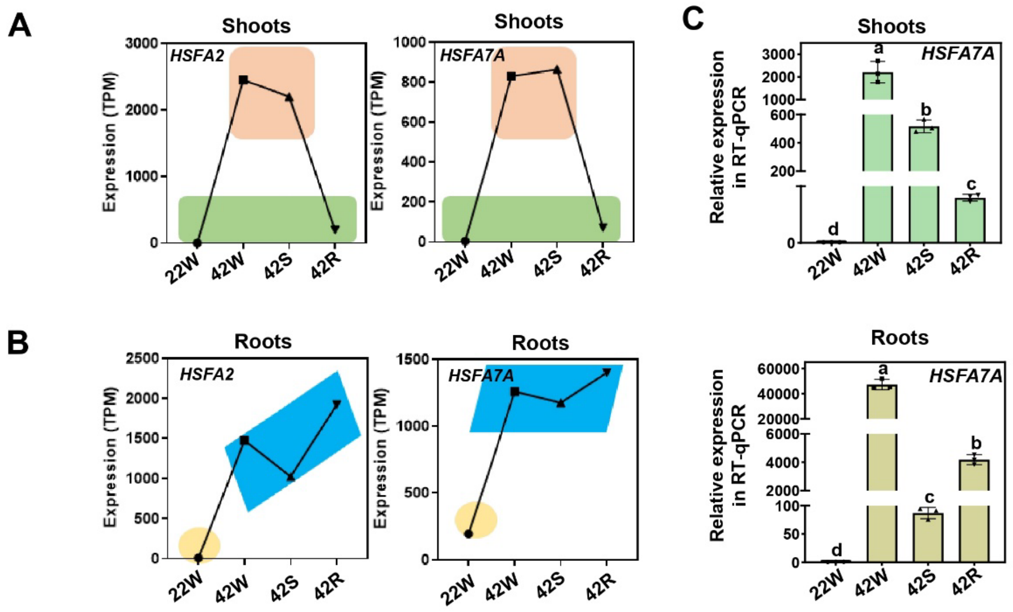

Supplemental Figure S1 The expression levels of *HSFA2* and *HSFA7A* were consistent with the results of our cluster dendrogram.

(A) Under conditions of high-temperature stress, the expression of *HSFA2* and *HSFA7A* in the 42R samples was comparable to that in the 22W samples (indicated by a green box), whereas the expression levels in the 42W and 42S samples were similar (as indicated by the pink box). **(B)** Under high-temperature stress, *HSFA2* and *HSFA7A* exhibited similar expression patterns in the 42W-R, 42S-R, and 42R-R samples (indicated by a blue frame), while the 22W-R samples displayed independent expression (denoted by a yellow circle). **(C)** *HSFA7A* expression was evaluated by RT-qPCR using *UBC21* as the internal control. Each data point represents the result from an individual biological replicate; error bars indicate the mean ± SE. Statistically significant differences are denoted by different lowercase letters ($P<0.05$, determined by a two-way ANOVA with Tukey's significant difference test).
