# Supplementary material for: Comparative Transcriptome Analysis of Arabidopsis Seedlings Under Heat Stress on Whole Plants, Shoots, and Roots Reveals New HS-Regulated Genes, Organ-Specific Responses, and Shoots-Roots Communication
Source: Int J Mol Sci. 2025 Mar 10;26(6):2478. doi: 10.3390/ijms26062478 (PMC11942352; doi:10.3390/ijms26062478)
Supplement: Supplementary file 1 [file ijms-26-02478-s001.zip › Additional file 1/Supplemental Fig. 2.pdf]

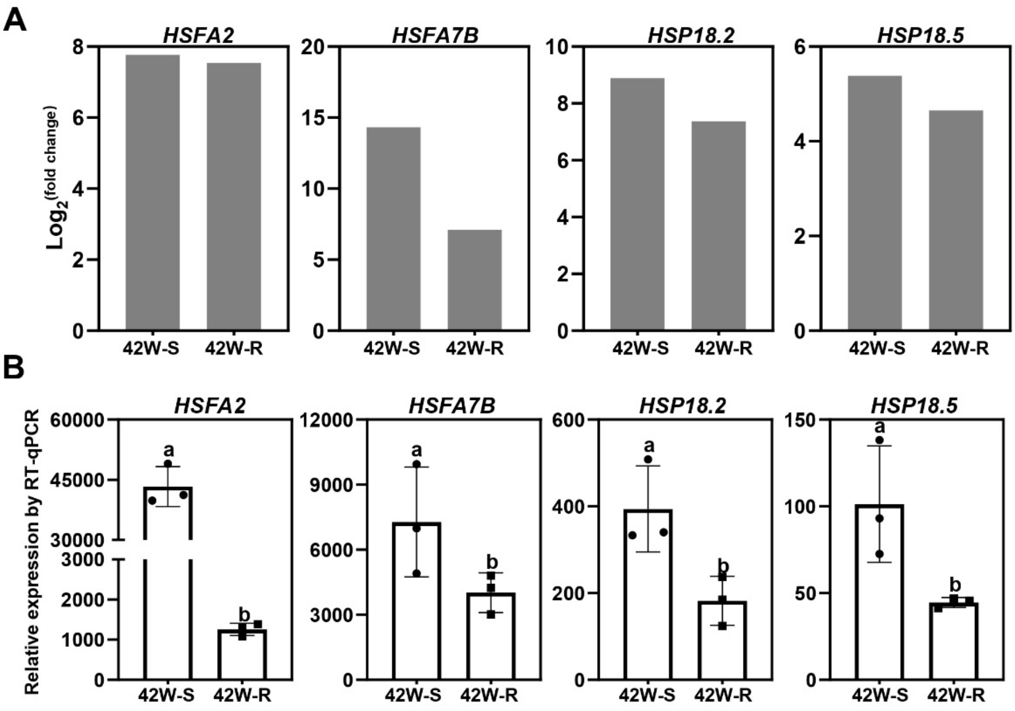

Supplemental Figure S2 Our RT-qPCR results were consistent with our RNA-Seq data regarding the expression of HS marker genes in the 42W-S/R samples.

(A) Marker gene expression based on our RNA-Seq data. **(B)** The relative expression levels of *HSFA2*, *HSFA7A*, *HSP18.2*, and *HSP18.5* are shown. *UBC21* was used as the internal control. Each dot represents the result from one biological replicate; error bars indicate the mean ± SE. Statistically significant differences are indicated by different lowercase letters ($P<0.05$, two-way ANOVA with Tukey's significant difference test).
