# Supplementary material for: Comparative Transcriptome Analysis of Arabidopsis Seedlings Under Heat Stress on Whole Plants, Shoots, and Roots Reveals New HS-Regulated Genes, Organ-Specific Responses, and Shoots-Roots Communication
Source: Int J Mol Sci. 2025 Mar 10;26(6):2478. doi: 10.3390/ijms26062478 (PMC11942352; doi:10.3390/ijms26062478)
Supplement: Supplementary file 1 [file ijms-26-02478-s001.zip › Additional file 1/Supplemental Fig. 3.pdf]

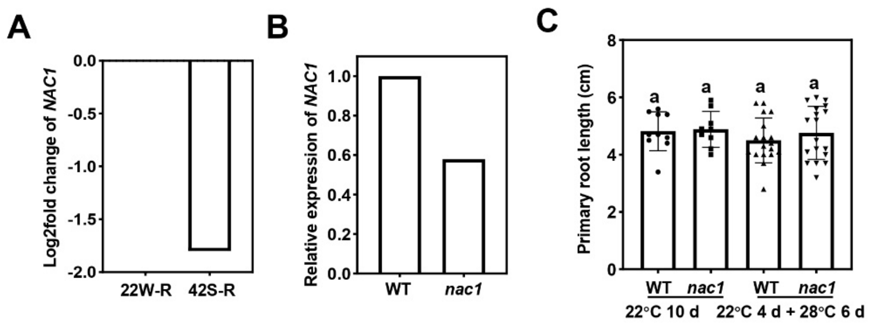

Supplemental Figure S3 *nac1* is a knock-down mutant and displays normal primary root length under HS at 28°C.

(A) *NAC1* expression based on our RNA-Seq data. **(B)** Identification of the decreased transcript level in the *nac1* mutant by RT-qPCR. **(C)** Quantification of the primary root length.
