# Supplementary material for: Comparative Transcriptome Analysis of Arabidopsis Seedlings Under Heat Stress on Whole Plants, Shoots, and Roots Reveals New HS-Regulated Genes, Organ-Specific Responses, and Shoots-Roots Communication
Source: Int J Mol Sci. 2025 Mar 10;26(6):2478. doi: 10.3390/ijms26062478 (PMC11942352; doi:10.3390/ijms26062478)
Supplement: Supplementary file 1 [file ijms-26-02478-s001.zip › Additional file 1/Supplemental Fig. 4.pdf]

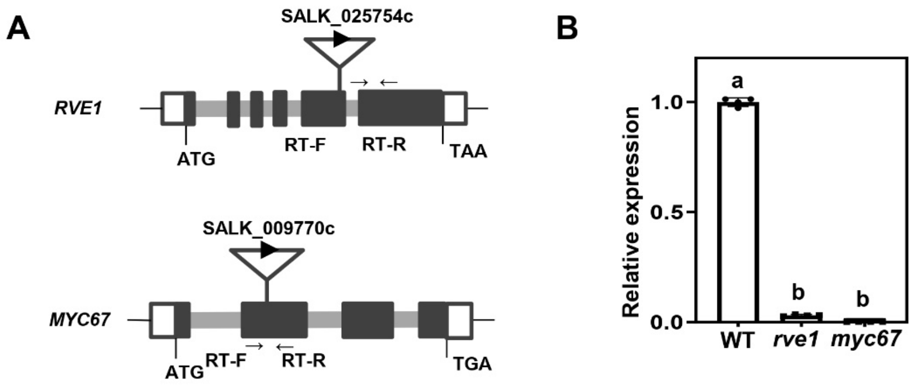

Supplemental Figure S4 *rve1* and *myc67* are knock-out mutants.

(A) Schematic representation of the T-DNA insertions in *RVE1* and *MYC67*. Black, gray, and white closed boxes indicate exons, introns, and untranslated regions, respectively. **(B)** The transcript levels in the *rve1* and *myc67* mutants were down-regulated based on RT-qPCR. *UBC21* was used as the internal control.
