# Supplementary material for: Comparative Transcriptome Analysis of Arabidopsis Seedlings Under Heat Stress on Whole Plants, Shoots, and Roots Reveals New HS-Regulated Genes, Organ-Specific Responses, and Shoots-Roots Communication
Source: Int J Mol Sci. 2025 Mar 10;26(6):2478. doi: 10.3390/ijms26062478 (PMC11942352; doi:10.3390/ijms26062478)
Supplement: Supplementary file 1 [file ijms-26-02478-s001.zip › Additional file 1/Supplemental Table 1.pdf]

Supplemental Table S1. Summary of our RNA-Seq read mapping results

Sample ID	Raw reads	Mapped reads		Unique mapped reads	
		Number	% Mapped	Number	% Mapped
22W-S1	40597938	39585557	97.51	37958898	93.50
22W-S2	54951140	54358590	98.92	53194166	96.80
22W-S3	48763468	48086230	98.61	47076225	96.54
22W-R1	38148592	37024394	97.05	35973297	94.30
22W-R2	44677562	44068442	98.64	43247573	96.80
22W-R3	53838662	53065558	98.56	52145751	96.86
42W-S1	47256926	46279111	97.93	44438056	94.04
42W-S2	54824912	54244595	98.94	53280354	97.18
42W-S3	44827068	44297772	98.82	43398955	96.81
42W-R1	45821672	44806935	97.79	43546904	95.04
42W-R2	56548938	55844423	98.75	54887888	97.06
42W-R3	41828958	41309362	98.76	40465080	96.74
42S-S1	45620402	44669353	97.92	42925966	94.09
42S-S2	45023222	44507615	98.85	43703345	97.07
42S-S3	44764562	44246286	98.84	43370996	96.89
42S-R1	41511000	40516723	97.60	39615373	95.43
42S-R2	38174790	37693804	98.74	36901419	96.66
42S-R3	37898066	37389932	98.66	36713119	96.87
42R-S1	47375584	46336086	97.81	44605276	94.15
42R-S2	46908432	46356802	98.82	45551203	97.11
42R-S3	47405034	46790129	98.70	45936928	96.90
42R-R1	37058442	36151959	97.55	35243410	95.10
42R-R2	46598562	45984072	98.68	44993747	96.56
42R-R3	41690196	41136489	98.67	40319093	96.71
Total	1092114128	1074750219	98.41	1049493022	96.10
