# Supplementary material for: Comparative Transcriptome Analysis of Arabidopsis Seedlings Under Heat Stress on Whole Plants, Shoots, and Roots Reveals New HS-Regulated Genes, Organ-Specific Responses, and Shoots-Roots Communication
Source: Int J Mol Sci. 2025 Mar 10;26(6):2478. doi: 10.3390/ijms26062478 (PMC11942352; doi:10.3390/ijms26062478)
Supplement: Supplementary file 1 [file ijms-26-02478-s001.zip › Additional file 1/Supplemental Table 2.pdf]

Supplemental Table S2. The expression levels of DEGs from the 42W-S and 42W-R samples that were identified as functioning in "plant hormone signal transduction"

	Plant hormone signal transduction	
	**Shoots**	**Roots**
**Abscisic acid**	*AIP1* (2.71)	*AIP1* (5.86)
**Auxin**	*SAUR35* (4.88)	*SAUR35* (3.21)
**Cytokinin**	*ARR6* (-1.57)	*ARR6* (2.13)
**Auxin/IAA**	*SAUR27* (1.98) *WES1* (1.42)<br>*SAUR6* (-2.17) *SAUR20* (-1.97)<br>*SAUR14* (-1.94)<br>*SAUR16* (-1.85) *SAUR49* (-1.77)<br>*SAUR50* (-1.46)<br>*DFL2* (-1.35) *AFB1* (-1.31)	*AUR3* (1.96)
**Abscisic acid**	*SNRK2.3* (1.93) *ABI1* (1.91) *PYL9* (1.86)<br>*ABI2* (1.60) *PYL7* (1.56)<br>*PYL6* (-4.01)	*HAI1* (2.48)<br>*PYL4* (-2.28)
**Gibberellic acid**	*GAI* (-2.34) *GAL1* (-2.30)	
**Cytokinin**	*ARR4* (-1.32)	*ARR7* (2.01) *ARR5* (1.42)<br>*ARR9* (1.38)
**Brassinosteroid**	*BIN1* (-1.18)	
**Salicylic acid**	*OBF4* (-1.10)	

Observed changes in expression are shown in terms of the Log2FC from up-regulation (purple) to down-regulation (blue).
